# Global insights into food fraud from location‐based analysis: food adulteration in Turkey

**DOI:** 10.1002/jsfa.14302

**Published:** 2025-05-05

**Authors:** Murat Kavruk, Tuğçe Nur Balcı, İrem Çağla Özel, Veli Cengiz Ozalp, Ali Aydın

**Affiliations:** ^1^ Department of Medical Biology and Genetics, Faculty of Medicine Istanbul Aydın University İstanbul Turkey; ^2^ Department of Nutrition and Dietetics, Faculty of Health Sciences Atilim University Ankara Turkey; ^3^ Department of Medical Biology, School of Medicine Atilim University Ankara Turkey; ^4^ Department of Food Hygiene and Technology, Faculty of Veterinary Medicine Istanbul University‐Cerrahpaşa Istanbul Turkey

**Keywords:** food safety, food fraud, economically motivated food adulteration, Turkey

## Abstract

**Background:**

Food fraud and adulteration pose critical global challenges impacting economic stability and public health. This study examines the prevalence and characteristics of food fraud incidents in Turkey, an international player in the food supply chain. Controls carried out from production to consumption reveal many fraudulent events worldwide.

**Results:**

Data collected by the Ministry of Agriculture and Forestry of Türkiye from 2012 to 2022, covering 4007 incidents and 7180 specific cases of adulteration, form the basis of this analysis. The study categorizes food fraud by region, product group and type of fraud, revealing trends and patterns. Key findings indicate a higher incidence of fraud in milk, meat and vegetable oil products, including the detection of drug‐based adulteration having potential for serious health consequences.

**Conclusion:**

At most importance, we demonstrated the importance of risk‐based food inspections and the development of new detection technologies to enhance food safety. The results are fundamental for more effective food inspections in terms of risk‐based conformity assessment approaches or developing new methods, devices and analysis kits in terms of scientific and technological approaches. Still, they can also significantly improve future food safety measures. These insights are aimed at informing global food safety strategies and policymaking, contributing to a safer and more transparent food supply chain. © 2025 The Author(s). *Journal of the Science of Food and Agriculture* published by John Wiley & Sons Ltd on behalf of Society of Chemical Industry.

## INTRODUCTION

Food fraud is an umbrella term covering the intentional and economically motivated actions that cause a discrepancy between a product itself and the product claims intending to deceive the consumer.^1^, ^2^ Along with the industrialization of food manufacturing, food fraud and adulteration have become one of the leading global problems.^2^, ^3^ Producers usually commit food fraud for financial gain by deceiving their consumers or customers, also called economically motivated adulteration.^4^, ^5^ There are different classifications of food fraud from the perspective/working principle of an organization, like RAFF, EMA or HorizonScan.^6^, ^7^ Although there are some variations in these classifications, substitution, dilution and artificial enhancement are the main adulterations that can only be detected via laboratory activity.

Authorities and researchers have worked on databases, systems and strategies to detect and monitor food fraud and adulteration incidents. There are several databases (either pay‐to‐use or free) for monitoring food fraud, such as Rapid Alert System for Food and Feed (RASFF), Medical Information Systems (MEDISYS), FADB‐China and HorizonScan.^8^, ^9^ At the national and international levels, the presence and potential of such databases are crucial in developing strategies. Some of these detection/tracking/prediction strategies can be observed in the literature since food fraud and adulteration studies have shown an increasing trend over the past two decades.

The trend of scientific research can be plotted using a Boolean operator‐mediated search, ‘food fraud OR food adulteration’, in the ScienceDirect database, illustrated in Fig. S1. The scientific community is increasingly interested in food fraud, including method development, strategy development, traceability systems and case studies. Zhang and Xue^10^ depicted the situation of food fraud in China's domestic market by analyzing the economically motivated food fraud and adulteration cases featured in media reports. The data represented the region, fraud type, contaminants and food source. A more recent study by Bouzembrak *et al*. analyzed and predicted the food fraud incidents of the MedISys portal of European Media Monitor based on media reports.^8^ Such strategies can provide new insights for battling against economically motivated food fraud. Another research focused on Brazil and analyzed food fraud and adulteration data from academic journal reports. Food fraud and adulteration data were summarized in food groups, fraud type, contaminants, principal analysis methods, region and year.^11^ Researchers also analyzed the EU RASFF data from Poland on the adulteration of cereals and bakery products and from Finland, examining the patterns of food fraud and adulterations.^12^, ^13^ Another comprehensive study was conducted for Austria, focusing on identifying high‐risk products via analyzing the official food control dataset. The study successfully revealed the trend in the meat industry.^14^

As an essential agricultural exporter and importer in the international food chain, Turkey has ongoing food fraud and adulteration in its food industry and exports; however, there is no comprehensive study about the economically motivated food adulteration status in Turkey.^15^ The only literature that can be noted is the congress proceeding that our team presented as a preliminary study to attract attention to this lack of knowledge at the national level.^16^ Also, the lack of efficiency in the food fraud databases for monitoring food fraud and adulteration and the lack of systematic data analysis allow room for fraudulent activities.^17^ From this perspective, aggregation of publicized data by the Ministry of Agriculture and Forestry of Türkiye was accomplished for analyzing and evaluating food fraud data in terms of region, product group, fraud type, fraud content and trends aiming to help researchers, risk‐based quality systems and policy‐makers.

## MATERIALS AND METHODS

### Acquisition of data

Food fraud incidents data of Turkey have been publicized by the Republic of Türkiye Ministry of Agriculture and Forestry (formerly named the Ministry of Food, Agriculture and Livestock) between 2012 and 2022 in the announcements section of its official website (tarimorman.gov.tr). In addition, due to short announcement periods on the official website (about one month), lists of fraudulent firms and products from previous years were also collected from websites directly referencing the official website (such as tarimdanhaber.com). In this period, 23 announcements were made listing 4007 incidents determined by 7180 fraudulent detections, the details of which are given in Table S1.

The official authorities determined the incidents as a result of 10 576 422 field inspections resulting in 166 461 administrative fines and 1670 criminal complaints listed yearly based on Table S2.^18^ The difference between the announcements and administrative fines is that the announcements encompass only the intended food quality problems that laboratory methods have proved.

Ministry‐sourced official data have been chosen to analyze food fraud in Turkey for several reasons. First, these lists were created after the nationwide inspections of the Ministry, resulting in the most comprehensive data regarding the diversity of locations, food types and producers/sellers. Second, results were publicized after series of analyses by validated (accredited against TS EN ISO/IEC 17025) and Ministry‐approved laboratories (41 public and 101 private)^19^ as additional control of the samples in the case of positive results for food fraud, making the data highly trustable. The geographical distribution of these laboratories is mapped in Fig. S2. Finally, these lists have the same data format regarding data grouping and detail, allowing easy comparison and trend analysis.

### Analysis method

The data in the lists are composed of four columns initially encompassing the name of the firms with their addresses (which were included in this study only in terms of the frequency and legal status of the firms by excluding the names), name of the food and detected improper ingredient/analyte and brand of the product (also included in this study only as frequency of occurrence). Following the collection and concatenation of the lists, additional data were incorporated to support and deepen the analysis. The number of variables was expanded by derivatizing the original data: the legal status of the firms, city‐based location of the firms, type of food, type of fraudulent ingredient and detected ingredient. In addition, apart from the incidents listed, the number of detected ingredients/parameters were also counted and considered to understand the technical challenges (mentioned as ‘occurrences’ in the study). Data analysis was divided into four main categories, each aiming for an input for either conformity assessment or technological studies: location‐based, producer‐based, product‐based and fraud‐based representation. All data were concatenated in MS Office® Excel® program, then pivoted, graphed and mapped accordingly and analyzed in the results section.

## RESULTS AND DISCUSSION

### Region‐based analysis

**Figure 1:**
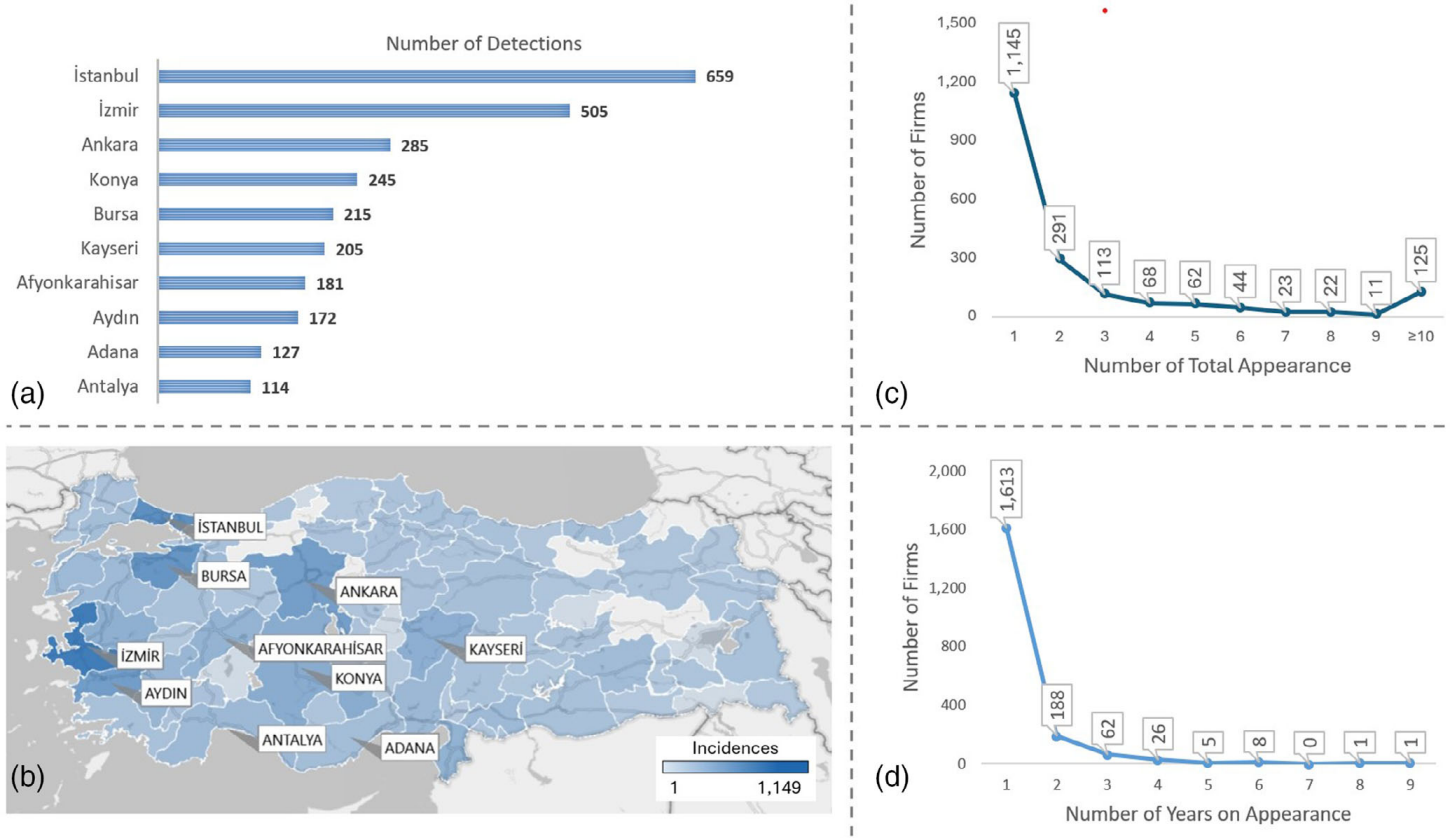
Region‐based and producer‐based distribution of publicized food fraud incidents in Turkey. (a) Number of incidents in the top 10 cities of the country. (b) Relative densities of incidents per city showing the concentrated areas and country‐wide distribution of food fraud detection. (c) Total number of occurrences and (d) number of years the same firm had been listed.

Location‐based analysis was the first approach to the food fraud data of Turkey to understand the distribution and any possible correlation in the country to detect the source of risks, optimize risk management and ensure a safe food chain.^20^ According to the analysis, the top 10 of 81 cities encompassed 68% of all incidents in the country. While 2708 of 4007 food fraud incidents had been detected in those 10 cities (270 incidents per city), 1299 had been detected in the remaining 71 cities (18 incidents per city) (Fig. 1(a)). At this stage, it is essential to note that the country's top 10 cities are major food production sites. However, as in the case of Balıkesir, one of the centers of food production, the correlation is not strict. Many of Central Anatolia's cities focus on large‐scale, low‐processed food production (like poultry and egg production), the main ingredients of other further processed foods. On the other hand, the number of food fraud incidents detected by the Ministry has a similar density to the population of these cities. Since the inspections also covered food service sites (such as restaurants), a wider range of food chain‐related facilities would be under inspection in more industrialized and populated cities.

Another question related to the location of the fraudulent food data was whether the presence of analysis laboratories in a city could affect the food fraud incidents. A comprehensive correlation analysis may not be conducted due to the restrictions of knowledge about all the in‐house laboratories, including their validation range and technical precision. This is a crucial lack of information in Turkey, a fertile research area. However, as mapped in Fig. S2, the regional distribution of Ministry‐authorized inspection laboratories gave us a similar distribution of fraudulent incidents in Turkey, also mapped in Fig. 1(b).

### Producer‐based analysis

According to the data, 1904 firms are mentioned in the Ministry's lists, with an average of 2.1 food fraud cases occurring for each firm involving fraudulent activity. Table 1 summarizes the data in the lists concerning the legal status of the firms. According to the names of the firms, legal statutes were determined as to whether they were separate legal entities (corporate) or private companies. Among the 1904 firms, the legal status of 946 could not be deduced, and thus tagged as ‘unknown’. For the remaining firms and their appearances in the fraudulent lists, as independent legal entities, companies had a 1.4 occurrences per firm ratio, while person(s)‐controlled private companies had a much higher value of 2.9 occurrence per firm ratio of appearance in the fraudulent lists. It is possible to speculate that there is a higher potential for food fraud in private companies. Although there is a need for additional data like the number and distribution of products or systems (like ISO 22000 or FSSC 22000) certification in these firms, it would be possible to conclude that there is a firm size‐ and/or production volume‐dependent tendency for economically motivated food fraud. Such speculations are also catalyzers of further studies in this field.

**Table 1:** Number of firms according to their legal status and occurrences in the lists

Legal status	Number	Occurrences	Ratio
Corporation	148	206	1.4
Private	810	2326	2.9
Unknown	946	1475	1.6
Total	1904	4007	2.1

The second analysis related to the firms was conducted based on the total and year‐based occurrences of these firms. The following results were compiled by deriving the graphs in Fig. 1(c) from the original data. A total of 1145 of 1904 firms (60%) had only been involved in the food fraud lists once. The remaining 759 firms (40%) had been listed more than once. The year‐based occurrences graph gave us a more substantial clue for the persistence of firms to economically motivated food fraud actions and the effectiveness of inspection and surveillance activities. A total of 1613 of 1904 firms (85%) had been publicized only in 1 year between 2012 and 2022 (Fig. 1(d)). Given that the first and final lists of the year may be the results of consecutive years, some of the two‐year occurrences are misleading; one‐year occurrences of firms would probably have a higher ratio than expressed in the lists. Such results led us to conclude that market surveillance and inspection – as a form of conformity assessment – are more effective than laboratory infrastructure in combat against economically motivated food fraud since, without the synergistic effect of market surveillance, laboratories would be insufficient to ‘target’ the fraud. However, both the accessibility and deterrence of market surveillance and inspection were enough for the food fraud cases in Turkey.

The final investigation related to producers was derived from the previous analysis. Product groups that appeared in the top 10 firms listed are summarized in Table 2. Five so‐called ‘persistent’ firms (four in the top five) are against consecutive and widespread market surveillance and inspection and operate in the milk and milk products sector. Four of the remaining firms are from the vegetable oils sector. Two firms are involved in more than one subsector, according to the Ministry's classification. When the same analysis was applied to all of the firms, 59 of 1904 were determined to be listed in more than one product group (data not shown).

**Table 2:** Firms that appeared most in the Ministry fraudulent product lists

Product groups/top 10 firms	1	2	3	4	5	6	7	8	9	10	Total
Apiculture products	—	—	—	12	—	—	—	—	—	—	**12**
Herbal, tea and coffee products	—	—	—	—	—	1	—	—	—	—	**1**
Vegetable oils	—	—	60	—	—		34	31	30	—	**155**
Chocolate and cacao products	—	—	—	—	—	17	—	—	—	—	**17**
Soft drinks	—	—	—	—	—	3	—	—	—	—	**3**
Milk and dairy products	63	60	—	30	40		—	—	—	29	**222**
Supplements	—	—	—	—	—	17	—	—	—	—	**17**
**Total**	**63**	**60**	**60**	**42**	**40**	**38**	**34**	**31**	**30**	**29**	**427**

### Product‐based analysis

**Figure 2:**
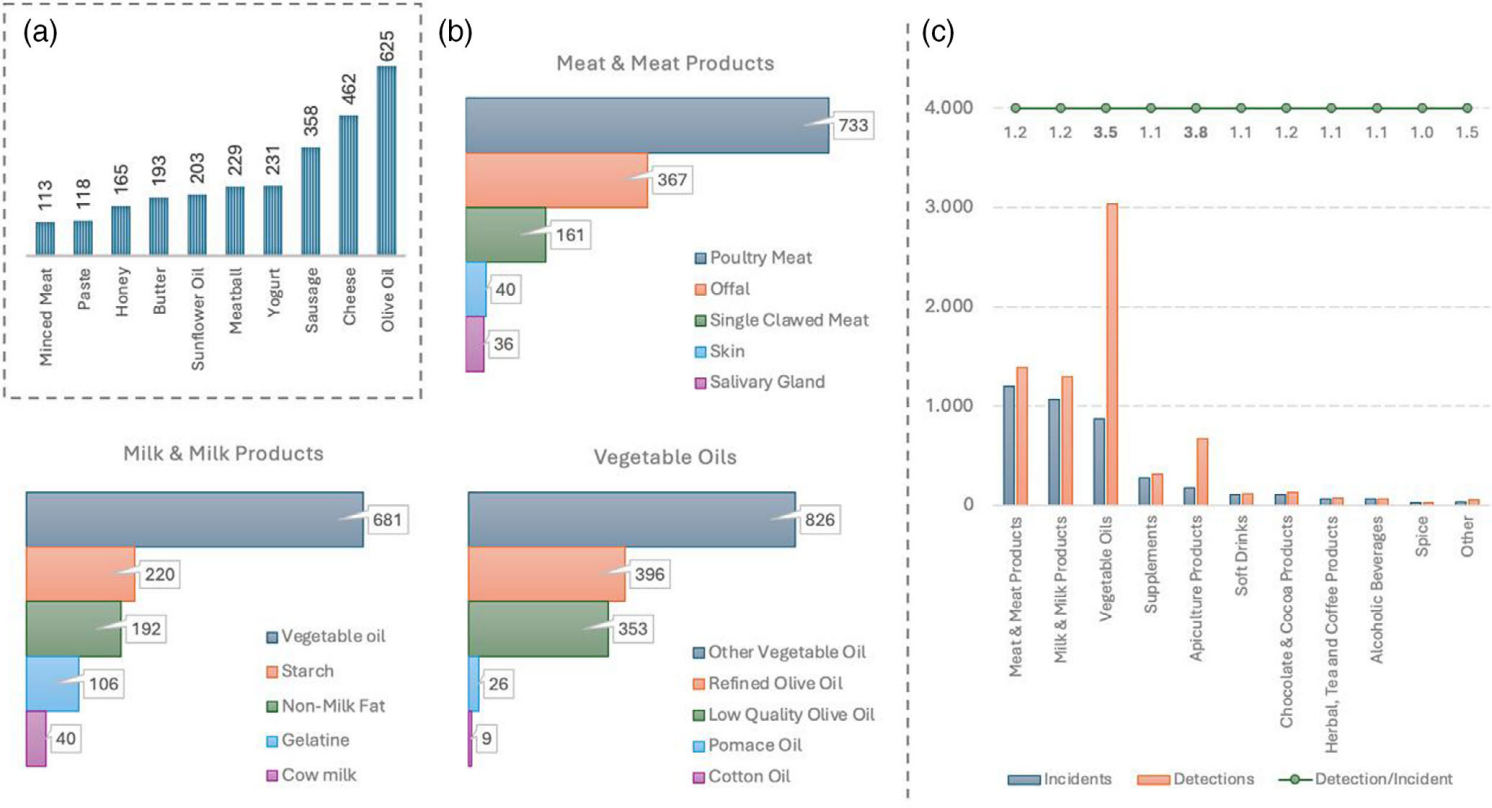
Major focused products and product groups. (a) Top 10 products from the fraudulent activity lists, commercial product names were deconstructed and types of food names were sorted. (b) Fraudulent details of major product groups. The top five fraudulent ingredients are graphed for each product group. (c) Relationship between fraudulent incidents and detections. Average number of detected fraudulent ingredients/processes for each product group incident. The ratio between these numbers is also mentioned in the graph. Notice that the ratio increases for mainly non‐processed foods like vegetable oils and apiculture products.

Understanding which products are prone to fraudulent activities is another crucial parameter in analyzing food fraud events in Turkey. In doing so, one of the problems is that there are various product types (e.g. cheese); however, from the fraud/adulteration perspective, they are all the same product. Thus, major food types utilized in fraudulent activities were determined using the Ministry's declassified product names. As listed in Fig. 2(a), between 2012 and 2022, olive oil, cheese, sausage, yogurt and meatballs were the top five products that were victims of economically motivated food fraud activities in Turkey. From these top 10 product types, milk, meat and vegetable oil products are highly dominant, aligning with the literature.^21^ This trend is also maintained in the total list based on product groups. Throughout the 10 years, meat and meat products, milk and milk products and vegetable oils were revealed as the primary food product groups vulnerable to food fraud. These three product types encompassed 78% (3199 of 4007) of all incidents. Due to their majority in the product groups, further analysis is needed for meat and meat products, milk and milk products and vegetable oils. Sorted and graphed in Fig. 2(b) are the top five fraudulent activities conducted in food types belonging to these groups. Substitution and dilution of meat products, including poultry meat, milk products with vegetable oil and olive oil with other vegetable oils were the most common fraudulent activities detected.

The Ministry publicized product types of the 4007 food fraud incidents in the lists, and these food types are also detailed in Table 3. Consistent with the worldwide data, meat and meat products were the most reported frauded food products.^7^ A maximum number of year‐based detection for each product type is indicated in bold in the table. Most food types reached their maxima in 2019, parallel with the number of publicized incidents in Table S1. Given that there was no publication in 2017 and only one in 2018, the cumulation of ready‐to‐publicized data may result in that increase in 2019. However, the so‐called persistence and dominance of these top three product groups are still reasonable since they represent the main diet ingredients of Turkish cuisine. On the other hand, the market surveillance and inspection procedure for apiculture products seemed to decrease fraudulent cases throughout the years. It is important to note that, independent of this cumulative effect of 2019, other product groups reached their peak in 2020 (before the widespread impact of the COVID‐19 pandemic in the country), and some of these product groups (like soft drinks or tea products) were vulnerable to drug‐based adulteration such as sildenafil or sibutramine. New lists that will be published for more accurate risk‐based decisions must support such opinions and conclusions.

**Table 3:** Distribution and year‐based trends of fraudulent incidents according to product types. Bold numbers represent the highest number of incidence of each product group

Product group	2012	2013	2014	2015	2016	2018	2019	2020	2022	Total	Ratio
Meat and meat products	6	17	42	94	146	70	**464**	231	133	1203	30%
Milk and dairy products	35	69	56	102	129	62	**272**	184	156	1065	27%
Vegetable oils	‐	3	2	60	125	104	**298**	166	113	871	22%
Supplements	5	23	17	39	28	15	**57**	51	41	276	7%
Apiculture products	1	**48**	10	19	16	15	27	16	23	175	4%
Soft drinks	3	‐	5	16	11	6	**31**	26	13	111	3%
Chocolate and cocoa products	‐	‐	2	3	7	6	31	**40**	21	110	3%
Herbal, tea and coffee products	‐	‐	‐	1	‐	1	17	**25**	22	66	2%
Alcoholic beverages	‐	‐	‐	1	6	‐	1	**32**	23	63	2%
Spice	‐	‐	2	1	1	1	6	**10**	8	29	1%
Other	0	3	4	**9**	5	0	6	8	3	38	1%
Total	50	163	140	345	474	280	1210	789	556	4007	100%

For further analysis, incidents and detections (number of detected parameters used for understanding/concluding fraudulent activity) were graphed to visualize the detection challenge. Figure 2(c) shows that, apart from vegetable oils and apiculture products, fraudulent activities can be detected by mostly one laboratory result, which is represented in the figure as the ‘detection/incident’ ratio. This ratio is between 1.0 and 1.5 detected ingredient/process for most of the product groups since most of the commercial products are processed foods, making a challenge for laboratory methods. On the other hand, fraudulent activities on less‐processed foods like vegetable oils and apiculture products can be detected by either different methods or by looking at various parameters. This reality can be observed in the ‘detection/incident’ ratio of vegetable oils (on average, 3.5 different detections for each incident) and apiculture products (on average, 3.8 different detections for each incident).

### Fraud‐based analysis

The final analysis approach to the fraudulent food data concerns the types of fraud and different fraudulent ingredients utilized in food. The first investigation was conducted on the label claims made by the firms about their products in fraudulent foods. Some specifications about the products were derived from the names of the products (apart from the brands). Aiming to convince consumers, most of the claims were related to the type of cheese, the authenticity of olive oils or species of the animal/type of processing of meat and meat products, which are presented as a word cloud in Fig. S3. It is important to note that all of the declarations ‘epimedium’ and ‘ginseng’ matched sildenafil and its derivatives regarding fraudulent ingredients.

**Figure 3:**
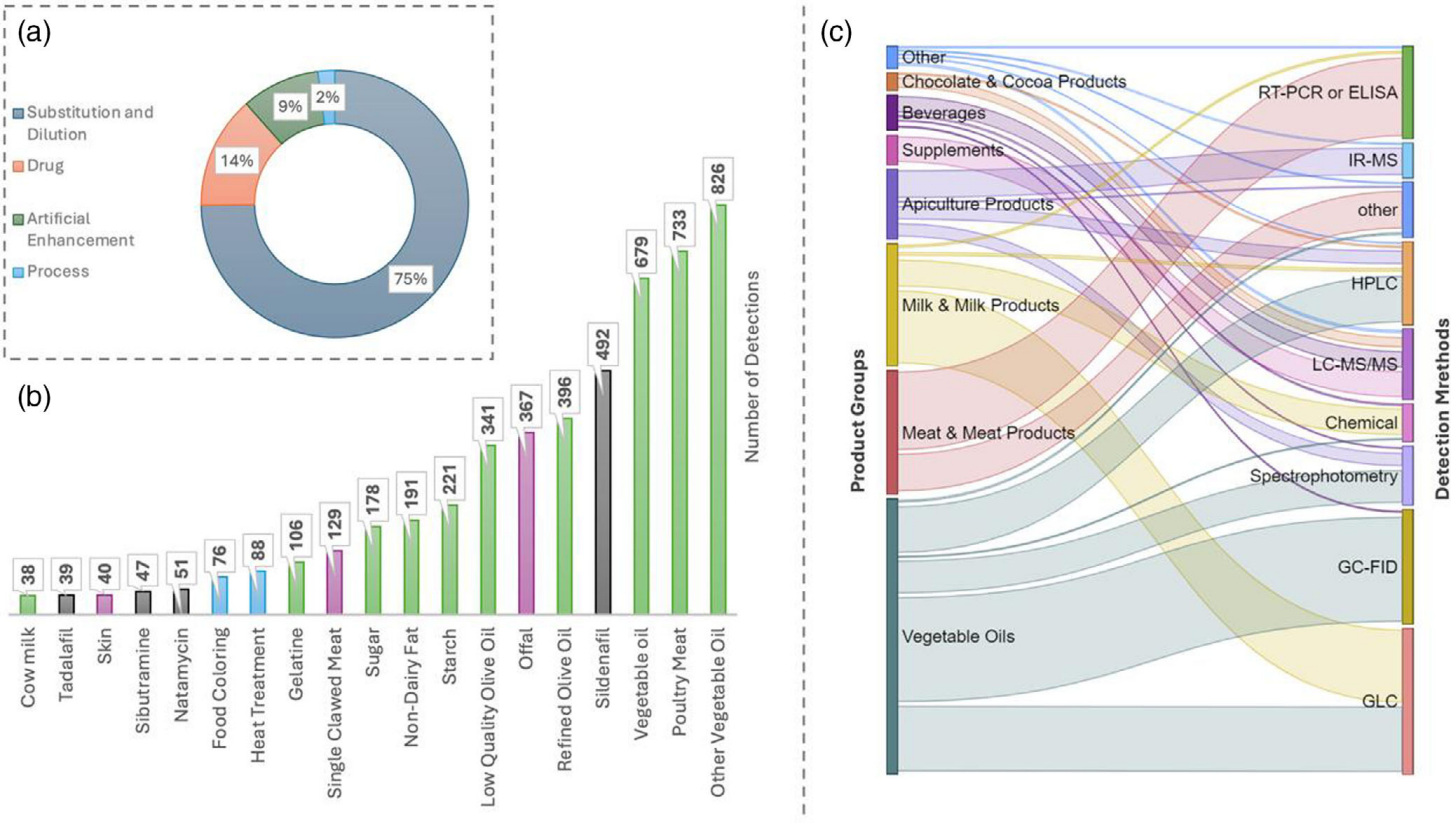
Analysis of fraudulent activities: (a) distribution of the detected food fraud types. (b) Primary fraudulent detections of ingredients/applications in listed incidents. Non‐declared food‐grade compounds (green bars), products of animal origin not suitable for human consumption according to legislation (purple bars), pharmaceuticals and drugs (black bars) and other types of fraudulent activities (blue bars) were stated. (c) Detection methods used by Ministry‐approved laboratories for different product groups.

The 10‐year period of cumulative fraudulent data showed that there are four main types of fraud mentioned in the lists of the Ministry which were subject of laboratory experiments: ‘substitution and dilution’ (adding an ingredient with less economic value than the original food to increase the content of the food), ‘drug’ (adding a pharmacologically active ingredient to mimic or boost natural form of the food or declared legal or natural alternative), ‘artificial enhancement’ (adding an ingredient to provide or gain the food the desired look/smell/texture artificially) and ‘process’ (handling of any type to mimic the product to its original form). Since the amount of fraudulent ingredients was not mentioned in the publicized data, it is not always possible to differentiate substitution from dilution. Thus, these food fraud types were discussed in the study for clarity. Another derivation from conventional food fraud classification was the separation of ‘drug’ from ‘artificial enhancement’. The addition of pharmacologically active substances to enhance food artificially must be counted more seriously than other food quality problems or artificial enhancement activities. Thus, tagging and tracking of food frauds with drugs would be meaningful. From this perspective, 4007 incidents of food fraud were distributed, and the majority of food fraud activity was found to be in the category of ‘substitution and dilution’ (75%) to increase the quantity of the product (Fig. 3(a)). The total number of fraud types (4315) was more than the total number of fraudulent incidents in the lists (4007) since, in some incidents, more than one type of fraudulent activity took place. Substances used in substitution and dilution, as well as types of processes for fraudulent activity, can be guessed and efficiently targeted in analyzing foods. However, artificial enhancement and drug‐based adulterations are both more challenging to detect and have higher potential for health risks. One of the difficulties is detecting the adulteration quantity for these two groups. Another difficulty is the diversity of enhancement substances and drugs. Apart from looking for ‘something’, the challenge is seeking ‘anything potential’ in the food samples. In analytical laboratory terms, omics‐oriented scientific and technological approaches (e.g. metabolomics) are crucial for conformity assessment studies in Turkey to fortify food quality and control measures.

Adulteration by adding a pharmacologically active drug ingredient is the most alarming in food fraud cases. According to detections, there are two important groups of drugs: sildenafil (and its derivatives) and sibutramine were found in food samples, and these two drugs were in the significant fraudulent ingredients (sildenafil in the top five) as presented in Fig. 3(b). Primary ingredients (and ‘heat treatment’) are listed concerning their occurrence number in the lists in the figure also. These ingredients are potential topics for new and on‐site detection method/device/biosensor development projects to gain an advantage over economically motivated food fraud tendencies. Thus, in‐house or outsourced quality control measures can be reviewed and improved based on the detections mentioned in the lists. Based on the methods used by Ministry‐approved laboratories, methods used and product groups were matched (Fig. 3(c)). While gas chromatography with flame ionization detection and gas–liquid chromatography are the main methods for detecting fraudulent activities, RT‐PCR or ELISA are mainly used for meat and meat products. According to the official declaration of food control laboratories (Istanbul was an example), the average time for giving results is 2.13 ± 1.65 days, most probably due to laboratory management optimization. With the concatenation of sample logistics, sample preparation and testing procedures, there will always be a delay in taking action in fraud detection, creating challenges and technological opportunities in conformity assessment.

## CONCLUSION

The data and summaries presented in this study have the potential for comparing food fraud tendencies, the effectiveness of the conformity assessment activities and developing new technologies and strategies for preventive actions in other countries. System standards published by the International Standards Organization (ISO 22000:2018) or other third parties (FSSC‐22000 v6.0) dictate food safety through hazard control and mitigation of food fraud to prevent adverse health consequences of potential threats. The data deconstructed and visualized in this study will support risk‐based decisions. Considering its importance in the global food chain and attracting millions of international tourists annually, addressing the lack of food fraud analysis in Turkey is crucial. To our knowledge, this is the pioneer and most comprehensive study conducted for Turkey intended to guide policymakers, conformity assessment‐oriented organizations and researchers working on food analysis in the high‐priority and prevalent areas of food fraud and related preventive measures.

## FUNDING INFORMATION

This research received no specific grant from public, commercial, or not‐for‐profit funding agencies.

## CONFLICT OF INTEREST STATEMENT

The authors declare no conflict of interest.

## AUTHOR CONTRIBUTIONS

**Murat Kavruk:** Conceptualization, Methodology, Software, Formal analysis, Writing – original draft. **Tuğçe Nur Balcı:** Formal analysis, Writing – reviewing and editing. **İrem Çağla Özel:** Formal analysis, Writing – reviewing and editing. **Veli Cengiz Ozalp:** Writing – reviewing and editing, Supervising. **Ali Aydın:** Writing – reviewing and editing, Supervising.

## Supporting information


**Figure S1.** The number of peer‐reviewed research articles about food fraud and food adulteration retrieved from the Science Direct database. Search criterion: ‘food fraud’ OR ‘food adulteration’.
**Table S1.** The number of food fraud incidents publicized by the Ministry of Agriculture and Forestry of Türkiye. The detections column represents the number of different analytical findings indicating a fraudulent situation in the samples analyzed.
**Table S2.** Field inspections and legislative results conducted by the Ministry of Agriculture and Forestry of Türkiye.
**Figure S2.** Geographical distribution of authorized food control laboratories in Türkiye.
**Figure S3.** Food product descriptions in fraudulent incidents. The words written in a dark colour are the top five product specificity used in marketing.

## Data Availability

The data that support the findings of this study are available from the corresponding author upon reasonable request.
