# Supplementary material for: Global insights into food fraud from location‐based analysis: food adulteration in Turkey
Source: J Sci Food Agric. 2025 May 5;105(11):5833–9. doi: 10.1002/jsfa.14302 (PMC12260328; doi:10.1002/jsfa.14302)
Supplement: Supplementary file 1 — Figure S1. The number of peer‐reviewed research articles about food fraud and food adulteration retrieved from the Science Direct database. Search criterion: ‘food fraud’ OR ‘food adulteration’. Table S1. The number of food fraud incidents publicized by the Ministry of Agriculture and Forestry of Türkiye. The detections column represents the number of different analytical findings indicating a fraudulent situation in the samples analyzed. Table S2. Field inspections and legislative results conducted by the Ministry of Agriculture and Forestry of Türkiye. Figure S2. Geographical distribution of authorized food control laboratories in Türkiye. Figure S3. Food product descriptions in fraudulent incidents. The words written in a dark colour are the top five product specificity used in marketing. [file JSFA-105-5833-s001.docx]

**Supplementary Information**

**Figure S1.** The number of peer-reviewed research articles about food fraud and food adulteration retrieved from the ScienceDirect database. Search criterion: “food fraud” OR “food adulteration”.

**Table S1.** The number of food fraud incidents publicised by the Ministry of Agriculture and Forestry of Türkiye. The detections column represents the number of different analytical findings indicating a fraudulent situation in the samples analysed.

| Dates of Public Notifications | Number of Incidents | Number of Detections |
| --- | --- | --- |
| **2012** | **50** | **66** |
| April | 12 | 21 |
| November | 8 | 8 |
| December | 30 | 37 |
| **2013** | **162** | **275** |
| April | 66 | 128 |
| July | 45 | 58 |
| December | 52 | 89 |
| **2014** | **141** | **188** |
| February | 32 | 47 |
| April | 40 | 61 |
| October | 68 | 80 |
| **2015** | **345** | **563** |
| January | 45 | 65 |
| February | 49 | 79 |
| July | 186 | 299 |
| December | 65 | 120 |
| **2016** | **474** | **807** |
| September | 324 | 504 |
| December | 150 | 303 |
| **2018** | **280** | **565** |
| March | 280 | 565 |
| **2019** | **1,210** | **2,208** |
| January | 1,210 | 2,208 |
| **2020** | **789** | **1,520** |
| January | 386 | 781 |
| February | 99 | 182 |
| April | 55 | 96 |
| June | 136 | 253 |
| September | 113 | 208 |
| **2022** | **559** | **987** |
| March | 559 | 987 |
| **Total** | **4,010** | **7,179** |

**Table S2.** Field inspections and legislative results conducted by the Ministry of Agriculture and Forestry of Türkiye.

| Inspection Area | Year | Number of Inspections | Number of Administrative Fine | Criminal Complaint |
| --- | --- | --- | --- | --- |
| Food Production Areas | 2012 | 87,234 | 6,700 | 92 |
|  | 2013 | 112,719 | 5,218 | 71 |
|  | 2014 | 126,428 | 5,068 | 63 |
|  | 2015 | 138,981 | 4,974 | 53 |
|  | 2016 | 146,388 | 4,876 | 61 |
|  | 2017 | 174,379 | 5,575 | 85 |
|  | 2018 | 180,027 | 6,277 | 102 |
|  | 2019 | 193,430 | 5,726 | 100 |
|  | 2020 | 214,626 | 5,528 | 93 |
|  | 2021 | 216,829 | 5,808 | 71 |
|  | 2022 | 204,211 | 6,509 | 117 |
|  | **Total** | **1,795,252** | **62,259** | **908** |
| Food Selling Areas | 2012 | 187,648 | 6,276 | 28 |
|  | 2013 | 217,937 | 3,918 | 37 |
|  | 2014 | 251,327 | 4,030 | 19 |
|  | 2015 | 315,611 | 4,564 | 28 |
|  | 2016 | 391,149 | 5,090 | 30 |
|  | 2017 | 467,411 | 5,858 | 50 |
|  | 2018 | 469,280 | 6,537 | 51 |
|  | 2019 | 498,743 | 6,004 | 44 |
|  | 2020 | 603,813 | 5,975 | 49 |
|  | 2021 | 606,909 | 5,631 | 55 |
|  | 2022 | 590,513 | 7,616 | 74 |
|  | **Total** | **4,600,341** | **61,499** | **465** |
| Mass Consumption Areas | 2012 | 138,116 | 4,096 | 24 |
|  | 2013 | 183,597 | 2,864 | 24 |
|  | 2014 | 223,169 | 2,767 | 16 |
|  | 2015 | 280,270 | 3,432 | 11 |
|  | 2016 | 356,427 | 4,109 | 11 |
|  | 2017 | 412,065 | 5,126 | 46 |
|  | 2018 | 475,611 | 5,350 | 33 |
|  | 2019 | 523,823 | 4,698 | 30 |
|  | 2020 | 538,204 | 3,059 | 30 |
|  | 2021 | 554,447 | 2,914 | 20 |
|  | 2022 | 535,100 | 4,288 | 52 |
|  | **Total** | **4,220,829** | **42,703** | **297** |
| Total | 2012 | 412,998 | 17,072 | 144 |
|  | 2013 | 514,253 | 12,000 | 132 |
|  | 2014 | 600,924 | 11,865 | 98 |
|  | 2015 | 734,862 | 12,970 | 92 |
|  | 2016 | 893,964 | 14,075 | 102 |
|  | 2017 | 1,013,855 | 16,559 | 181 |
|  | 2018 | 1,124,918 | 18,164 | 186 |
|  | 2019 | 1,215,996 | 16,428 | 174 |
|  | 2020 | 1,356,643 | 14,562 | 172 |
|  | 2021 | 1,378,185 | 14,353 | 146 |
|  | 2022 | 1,329,824 | 18,413 | 243 |
|  | **Total** | **10,576,422** | **166,461** | **1,670** |


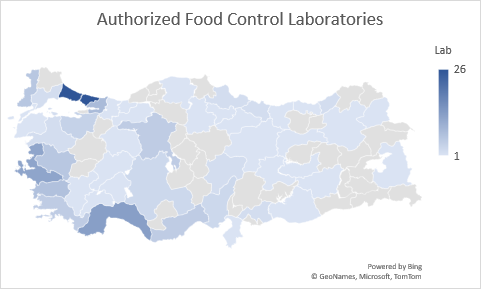


**Figure S2.** Geographical distribution of authorised food control laboratories in Türkiye.


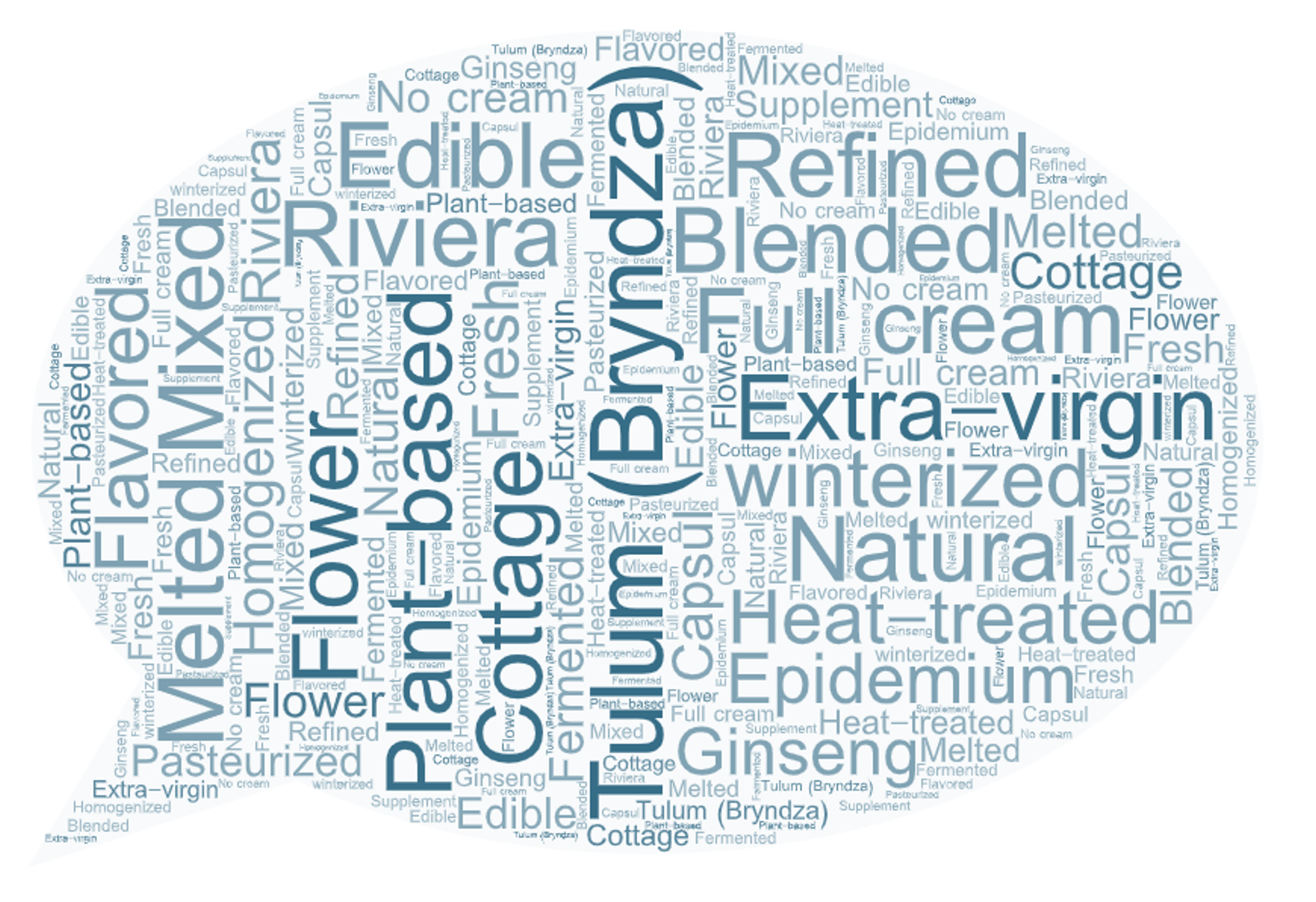


**Figure S3.** Food product descriptions in fraudulent incidents. The words written in a dark colour are the top five product specificity used in marketing.
